# Comparative genomics analyses of alpha-keratins reveal insights into evolutionary adaptation of marine mammals

**DOI:** 10.1186/s12983-017-0225-x

**Published:** 2017-08-02

**Authors:** Xiaohui Sun, Zepeng Zhang, Yingying Sun, Jing Li, Shixia Xu, Guang Yang

**Affiliations:** Jiangsu Key Laboratory for Biodiversity and Biotechnology, College of Life Sciences, Nanjing Normal University, Nanjing, 210023 China

**Keywords:** Marine mammals, Hair, α-keratin, Gene loss, Pseudogenization rate

## Abstract

**Background:**

Diversity of hair in marine mammals was suggested as an evolutionary innovation to adapt aquatic environment, yet its genetic basis remained poorly explored. We scanned α-keratin genes, one major structural components of hair, in 16 genomes of mammalian species, including seven cetaceans, two pinnipeds, polar bear, manatee and five terrestrial species.

**Results:**

Extensive gene loss and high pseudogenization rate of α-keratin genes were identified in cetaceans when compared to terrestrial artiodactylans (average number of α-keratins 37.29 vs. 58.33; pseudogenization rate 29.89% vs. 8.00%), especially of hair follicle-specific keratin genes (average pseudogenization rate in cetaceans of 43.88% relative to 3.80% artiodactylian average). Compared to toothed whale, the much more number of intact functional α-keratin genes was examined in the baleen whale that had specific keratinized baleen. In contrast, the number of keratin genes in pinnipeds, polar bear and manatee were comparable to those of their respective terrestrial relatives. Additionally, four keratin genes (K39, K9, K42, and K74) were found to be pseudogenes or lost uniquely in cetaceans and manatees.

**Conclusions:**

Species-specific evolution of α-keratin gene family identified in the marine mammals might be responsible for their different hair characteristics. Increased gene loss and pseudogenization rate identified in cetacean lineages was likely to contribute to hair-less phenotype to adaptation for complete aquatic environment. However, the fully aquatic manatee still remained the comparable number of intact genes to its terrestrial relative, probably due to its perioral bristles and bristle-like hairs on the oral disk. By contrast, similar evolution pattern of α-keratin gene repertoire in the pinnipeds, polar bear and their terrestrial relatives was likely due to abundant hair to keep warm when they went ashore. Interestingly, some keratin genes were exclusively lost in cetaceans and manatees, likely as a result of convergent hair-loss phenotype to inhabit completely aquatic environment in both groups.

**Electronic supplementary material:**

The online version of this article (doi:10.1186/s12983-017-0225-x) contains supplementary material, which is available to authorized users.

## Background

Marine mammals are specific groups with evolutionary histories that their separate terrestrial relatives returned to the ocean on separate occasions and adapted to living all or part of their life in the aquatic environment. The living groups of marine mammals are generally including the following five groups: pinnipeds (seals, sea lions, fur seals, and walruses), cetaceans (whales, dolphins, and porpoises), sea otters, sirenians (dugongs and manatees), and polar bears [1]. Despite their independent evolutionary origins, these clades of the marine mammals have developed a series of adaptations to full or part aquatic environments, including morphological (e.g. streamlined shape, paddle-like limbs and feet) and physiological features adaptations (such as superb diving skill, echolocation, the thickened blubber, and etc.) [2].

Hair emergence is one of the major innovations in the mammalian evolution. Hair plays a key role in protection from mechanical insults, facilitated homeothermy, sense the immediate surrounding, sexual dimorphism, attraction of mates and etc. [3, 4]. Similar to other mammals, marine mammals are characterized by the presence of hair coat but with different evolution pattern among them. In order to reduce drag in the water, cetaceans and sirenians lack of hair coat because both are completely aquatic though cetaceans have body hair temporarily at fetuses [5, 6]. In contrast, the walrus has lost much of their hair (fur) and are characterized by thick layers of blubber under the skin to keep warm due to spending considerable time on land [5]. The Weddell seal grows a thin fur coat around its whole body except for small areas around the flippers [5]. The polar bear’s fur consists of a dense layer of ‘under fur’ and an outer layer of guard hairs that appear white to tan but are actually transparent. The guard hair is 5–15 cm long over most of the bear’s body [7]. The sea otter, however, has an exceptionally thick and dense coat of fur although almost time living in the sea [8]. However, the genetic bases of hair evolution in these clades of marine mammals remain poorly explored.

Hair is a kind of strongly keratinized tissue and is mainly composed of alpha-keratins and keratin associated-proteins (KRTAPs) [9, 10], which are encoded by a large number ssof multigene families and arranged in clusters on chromosomes [9, 10]. Alpha-keratins and KRTAPs are fibre-reinforced structures consisting of intermediate filaments embedded in an amorphous protein matrix which can provide support for the stability and rigidity of epidermal cells and tissue morphology [11]. The KRTAPs, as well known KAPs, are unique to mammals. According to the amino acid composition, KRTAP was divided into two major groups: high/ultrahigh cysteine (HS) and high glycine-tyrosine (HGT) that are essential for the formation of rigid and resistant hair shafts with α-keratins [9, 12]. By contrast, the α-keratin gene family was divided into two categories, i.e. epithelial keratin genes and hair keratin genes. A total of 54 functional keratin genes were identified in the human genome, which can be divided into 28 type I and 26 type II genes [10, 11]. The type I keratins consist of 17 epithelial and 11 hair keratins while the type II members comprise 20 epithelial and 6 hair keratins. In the 37 epithelial keratins, nine are specifically expressed in the hair follicle. Thus, the nine genes and 17 hair keratins are collectively referred to as hair follicle-specific keratins, which are reported to control the growth and variation of hair [10]. Previous studies characterized KRTAP gene repertoire in 22 mammals and found that gene family repertoire expansion, contraction, and pseudogenization were related to hair diversities in mammals [13]. In addition, an increase rate of hair keratin genes loss and pseudogenization were examined in two cetacean species when compared with fur terrestrial relatives, which was suggested to be associated with cetacean hairless phenotype [14]. However, previous studies only determined in one or two cetacean species could not provide comprehensive insight into genetic basis of hair diversities in marine mammals. Thus, more marine mammals were added in the present study, including seven cetacean species, two pinniped species, polar bear as well as one manatee. We first scanned the α-keratin repertoire from the genomes of the 11 marine mammals, and compared with that of their terrestrial relatives to test if α-keratin gene diversities are related to their hair features. Moreover, we tested whether there was common genes loss along with pseudogenization in the full aquatic cetaceans and sirenians as response for convergent hair-loss phenotype. Specially, we also tested if baleen whales retained much more α-keratin genes than toothed whales due to its specific keratinized baleen. From all the analyses, we expected to address the underlying genetic basis of the different hair phenotype in marine mammals.

## Methods

### α-keratin and KRTAP genes scanning and identification

We first identified α-keratin gene repertoires in the cow (*Bos taurus*) (coverage 7×,Btau_4.6.1) from UCSC Genome Browser website (http://genome.ucsc.edu/) taking all the known α-keratin gene sequences of human [14] as queries using BLASTN and TBLASTN algorithm [15]. All exon/intron junctions follow the cannonical AG/GT rule of splicing [16]. Meanwhile, we combined the online websites GENEWISE prediction (http://www.ebi.ac.uk/Tools/psa/genewise/) to verifying the accuracy of the sequence. The putative α-keratin genes in the cow were then taken as queries to explore the α-keratin multigene family in the genome of 11 marine mammals, including bowhead whale (*Balaena mysticetus*, coverage 150×, http://www.bowhead-whale.org/downloads/), minke whale (*B. acutorostrata*, coverage 92×, BalAcu1.0, NCBI), sperm whale (*Physeter macrocephalus*, coverage 75×, Physeter_macrocephalus_2.0.2, NCBI), Yangtze River dolphin (*Lipotes vexillifer*, Lipotes_vexillifer_v1, coverage 115×, NCBI), killer whale (*Orcinus orca*, coverage 200×, Oorc_1.1, NCBI), bottlenose dolphin (*Tursiops truncatus*, coverage 30×, Ttru_1.4, UCSC), Yangtze finless porpoise (*Neophocaena asiaeorientalis asiaeorientalis*, unpublished data), Weddell seal (*Leptonychotes weddellii*, coverage 82×, LepWed_1.0, NCBI), Florida manatee (*Trichechus manatus latirostris*, coverage 150×, TriManLat_1.0, NCBI), Polar bear (*Ursus maritimus*, coverage 101×, UrsMar_1.0, NCBI), Pacific walrus (*Odobenus rosmarus*, coverage 200×, Oros_1.0, NCBI, Additional file 1). The orthologous α-keratin genes were also scanned from the genome of their respective terrestrial relatives, such as sheep (*Ovis aries*, coverage 142×, Oar_v3.1, NCBI), alpaca (*Vicugna pacos*, coverage 22×, Vicugna_pacos-2.0.1, NCBI), African savanna elephant (*Loxodonta africana*, coverage 7×, Loxafr3.0, NCBI), giant panda (*Ailuropoda melanoleuca*, coverage 60×, AilMel_1.0, NCBI) (Additional file 1). Type I and type II keratins have their unique flanking sequences, which can ensures maximum searching these genes in mammalian genomes. In addition, in order to obtain as much as possible of the keratin repertoire, all the newly annotated gene sequences were taken as queries in blast searches against their own genomes. Finally, all genes were checked to test whether their best hit was a α-keratin gene from NCBI genome database http://www.ncbi.nlm.nih.gov/blast/Blast.cgi using BLASTN algorithm [17]. All the identified α-keratin genes were separated into three categories: intact genes, incomplete genes (abbreviated to ic and pseudogenes (abbreviated to p), according to amino acid alignment and blast results. All the keratins were named according to the revised keratin nomenclature of human [10].

In addition, the published human [13] KRTAP genes were as queries to search unannotated genomic sequences of KRTAP genes in seal, walrus and manatee by using BLASTN algorithm [15]. All KRTAP genes have single exon in mammals. The homology fragment more than 30 bp was retrieved in the present study in order to scan the KRTAP gene repertoire as fully as possible. Like α-keratin genes, all the newly annotated KRTAP gene sequences were used as queries in blast searches against the own genomes. In the end all KRTAP genes were checked whether their best hit was a KRTAP gene from NCBI genome database.

### Phylogenetic inference for α-keratin genes classification

First, all nucleotide sequences were aligned using MUSCLE in MEGA6.05 [18] and checked by eye. Second, to classify type I and II keratin genes to their respective family, we reconstructed the phylogenetic tree under the GTR + G+ I model using Maximum likelihood (ML) by RAxML [19] and Bayesian inference (BI) by MrBayes 3.2.3 [20]. The ML tree evaluated the best tree for each cluster, and supported for the nodes obtained with 1000 replications. For the Bayesian analyses, two simultaneous independent runs were performed for 40 × 10^6^ iterations of a Markov ChainMonte Carlo algorithm, with six simultaneous chains, sampling every 1000 generations. In case of misplacement in phylogenetic trees, we will recheck their sequence for correction. Based on the sequence homology and phylogenetic relationship, we divided α-keratin genes into different subfamilies. Finally, we mapped α-keratin gene organization of each species on their genomes.

### Gene conversion and recombination detection

To detect whether the gene conversion and recombination presented among keratin gene family that grouped together in the same species rather than with the members of the same family, we used the RDP4 software [21] to detect gene conversion and recombination events using RDP, Geneconv, Bootscan, MaxChi and Chimaera with 1000 permutations and cut off *p* value of 0.001.

### Likelihood analysis of gene gain and loss

To estimate the average gene gain/loss rate and to identify gene families that have undergone significant size changes, we applied CAFÉ v3.0 [22], a tool for the statistical analysis of the evolution of the gene family size. CAFÉ v3.0 can be estimated gene gain and loss rate among α-keratin gene family, calculated ancestral states of gene family sizes for each node in the phylogenetic tree and identified the average expansion or contraction gene family on each branch. For the CAFÉ analysis, we reconstructed the ultrametric tree as a starting tree for Bayesian inference of tree topologies and node ages using Markov chain Monte Carlo (MCMC) in BEAST v1.8.1 [23]. We ran six independent MCMC, 3 × 10^8^ steps long under individual gene models previously selected for reconstruction. We then checked for the convergence and stationarity using Tracer v1.6 [23]. Finally, we extracted the maximum clade credibility tree for combined tree sets using TreeAnnotator v1.8.1 [23].

## Results and discussion

### α-keratin gene family related to hair features

**Fig. 1:**
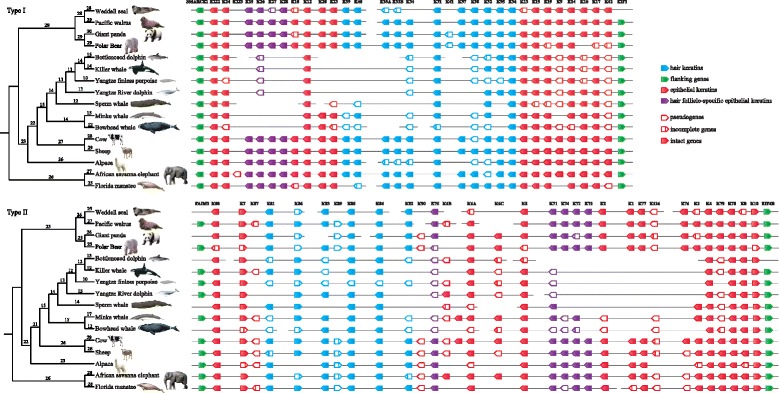
Genomic organization of α-keratin genes in 16 mammalian species according to phylogenetic tree, genomic position and blast results. The α-keratin genes name was based on that of the human. Connecting lines indicate α-keratin genes on the same chromosome/genomic scaffold. Direction of the arrows of each α-keratin gene indicate the direction in the contig. Filled figures: intact genes; empty figures: pseudogenes; empty figures with a vertical line: incomplete genes. In addition, all genes are divided into different colors: flanking genes (*green*); hair keratins (*blue*); epithelial keratins (*red*); hair follicle-specific epithelial keratins (*purple*). The numbers show the. Ancestral states of gene family sizes for each node in the phylogenetic tree

The advent of mainstream sequencing technologies has allowed the whole genomes of many mammals to be assembled, which provides possibility to explore the genetic basis of adaptation to their specific lifestyles at genome level. In the present study, we scanned the α-keratin repertoire from the genomes of 11 marine mammals (including seven cetaceans, two pinnipeds, polar bear and manatee) and compared with their terrestrial relatives (Table 1) to explore the genetic basis of hair diversity in marine mammals as response for their adaptations to aquatic lifestyle with different degrees. The genomic regions containing keratin gene families are reported to be conservative with special flanking sequences of two clusters, which facilitate to obtain the complete keratin repertoire in mammalian genomes. For example, type I keratins are flanked by SMARCE1 and EIF1 genes while type II keratins are flanked by FAIM2 and EIF4B genes (Fig. 1). However, we have not retrieved the FAIM2 gene in the 5′ flanking regions of five species, including Weddell seal, giant panda, bottlenosed dolphin, sperm whale, and bowhead whale (Fig. 1). Actually, the FAIM2 gene was identified in such five genomes but located in the different chromosomes with α-keratins. Similar phenomenon was also found in the EIF1and EIF4B that was not found in the flanking region of Weddell seal and bottlenose dolphin, respectively (Fig. 1). A total of 780 α-keratin genes were identified in the 16 mammalian species examined in our study, of which 383 belong to type I keratins, 397 belong to type II keratins.

**Table 1:** Numbers of α-keratin genes present in 16 mammalian species

Species	Total genes	Type I genes	Type II genes	Hair keratins	Hair follicle-specific epithelial keratins	Hair follicle-specific keratins
Weddell seal	54(1)[9]	28[5]	26(1)[4]	16[3]	9[1]	25[4]
Pacific walrus	58(2)[2]	29	29(2)[2]	18(1)	9	27(1)
Giant panda	58(2)[6]	30[2]	28(2)[4]	18(1)[2]	9[1]	27(1)[3]
Polar Bear	58(4)[9]	29[4]	29(4)[5]	19(2)[1]	9[1]	28(2)[2]
Bottlenosed dolphin	36(8)[7]	18(3)[4]	18(5)[3]	12(3)[3]	2(1)[1]	14(4)[4]
Killer whale	36(10)[2]	18(4)[2]	18(6)	11(3)	3 (2)[1]	14(5)[1]
Yangtze finless porpoise	36(16)[3]	18(8)[1]	18(8)[2]	12(9)[1]	3(3)	15(12)[1]
Yangtze River dolphin	34(8)	17(4)	17(4)	9(1)	3(3)	12(4)
Sperm whale	32(6)[3]	16(4)[2]	16(2)[1]	8(1)	1	9(1)
Minke whale	45(13)	19(4)	26(9)	13(5)	4(2)	17(7)
Bowhead whale	42(17)[7]	20(8)[3]	22(9)[4]	13(7)[2]	4(3)[1]	17(10)[3]
Cow	61(4)[2]	29(1)	32(3)[2]	18(1)[1]	9	27(1)[1]
Sheep	60(5)[3]	29	31(5)[3]	18[2]	9	27[2]
Alpaca	54(5)[7]	28(2)[3]	26(3)[4]	16(2)[4]	9	25(2)[4]
African savanna elephant	60(5)[3]	29(2)	31(3)[3]	18(1)[3]	9	27(1)[3]
Florida manatee	56(6)[4]	26(1)[2]	30(5)[2]	17(1)[1]	9(1)	26(2)[1]

Number of pesudogene and incomplete genes are represented in parenthesis and brackets, respectively

Hair keratins and hair follicle-specific epithelial keratins are collectively referred to as hair follicle-specific keratin

In contrast with the terrestrial mammals, the marine mammals have lost some α-keratins and are characterized with increased pseudogenization rate, likely related with their hair diversities in response to their living environment. Average of 44.27 α-keratin genes observed in the marine mammals has slightly differences to the terrestrial mammals which has averagely 58.8 α-keratins. Moreover, the pseudogenization rate of marine mammals (18.69%) is nearly three times of that of their terrestrial relatives (7.17%). In the case of the marine mammals, however, different evolution pattern of α-keratins among them might be responsible for their different hair characteristics. We found an average number of α-keratin genes in cetaceans of 37.29, which was significantly lower than that of artiodactylian species with an average number of 58.33. The number of α-keratin genes ranged from 32 in the sperm whale to 45 in the minke whale, while in artiodactylans it ranged from 54 in the alpaca to 61 in the cow (Table 1). The higher rate of pseudogenization rate was identified in cetacean species (average 29.89% compared to artiodactylans’ average of 8.00%). The cetacean species are nearly completely absent of hair coat with only a few bristle in order to reduce drag of swimming for adaptation to completely aquatic environment. There is evidence of an apparent reduction in the α-keratin gene repertoire and increased rate of pseudogenization in cetaceans, which may be associated with their hairless phenotype. For the fully aquatic manatee, body hairs are greatly reduced but with perioral bristles and bristle-like hairs on the oral disk that have specialized sensory and feeding function [24]. Accordingly, the manatee still remained the comparable number (56 in manatee vs. 60 in elephant) and rate of pseudogenization (8.33% in manatee vs. 10.71% in elephant) to its terrestrial relative, i.e. elephant (Table 1). By contrast, gene repertoires in the pinnipeds (56) and polar bear (58) were similar to that of panda (58) (Table 1). In addition, we detected 49 and 45 intact genes in pinnipeds and polar bear, comparable to that of the panda with 50 ones. Similar evolution pattern of α-keratin gene repertoire in the pinnipeds, polar bear and their terrestrial relatives is likely due to abundant hair to keep warm when they went ashore to mate, give birth, molt or escape from predators.

Further, we found that the gene gain and loss of α-keratin was consistent with the hair diversification of marine mammals. we employed the software CAFÉ to estimate the gene gain and loss of α-keratin typeI and typeII at each ancestral node in the phylogenetic tree. It was noted that loss of 6.5 genes in the ∼53–56 million years since the cetacean ancestor split from the cow/sheep clade. By contrast, the gain of five genes in the common ancestor of the cow/sheep. However, average one gene gain or lose in other marine mammals and their terrestrial relatives.

Hair follicle-specific keratin genes consisted of 9 hair follicle-specific epithelial keratin genes (K71-K75, K25-K28) and 17 hair keratins (K31-K40, K81-K86) expressed in hair follicle and hair which could directly affect hair growth [25]. A large number of diseases of hair were caused by mutations in hair follicle-specific keratin genes. For example, a mutation in K75 leads to loose anagen hair syndrome, which is characterized by easily pluckable hair [26]. In addition, the K71 mutation will make almost all types of mouse hair abnormal and easily fall off [27]. Subsequently, increased gene loss and pseudogenization rate were found to focus on hair follicle-specific keratin genes in cetacean lineages (Fig. 1). Only an average of 14 hair follicle-specific keratins were identified in cetaceans but with an average of 26.33 in artiodactylans. Most importantly, the averaged pseudogenized rate in cetaceans was 43.88%, 11 times higher than that in artiodactylans (3.80%). In summary, the unique gene loss and/or loss of their function in hair follicle-specific keratin genes might contribute to the hairless phenotype.

### Species-specific evolution of α-keratin gene repertoire

**Fig. 2:**
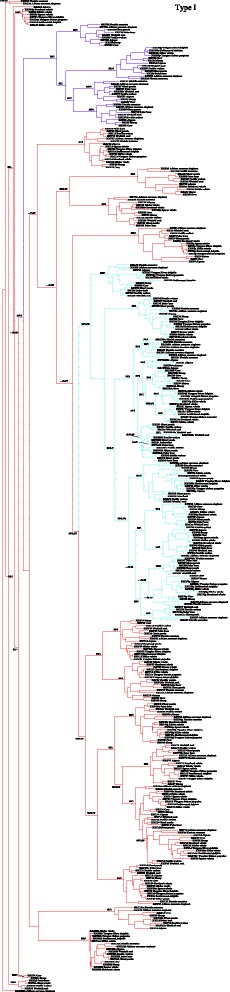
Phylogenetic tree of type I keratins. Maximum likelihood phylogram describing phylogenetic relationships among the type I keratin genes. Numbers above the nodes correspond to maximum likelihood bootstrap support values, and numbers below the nodes correspond to Bayesian posterior probabilities. Branches in *blue* indicated hair-type keratins, *purple* indicated hair follicle-specific epithelial keratins and *red* indicated epithelial keratins

**Fig. 3:**
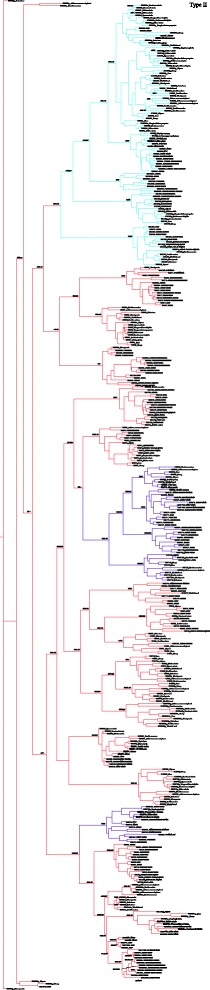
Phylogenetic tree of type II keratins. Maximum likelihood phylogram describing phylogenetic relationships among the type II keratin genes. Numbers above the nodes correspond to maximum likelihood bootstrap support values, and numbers below the nodes correspond to Bayesian posterior probabilities. Branches in *blue* indicated hair-type keratins, *purple* indicated hair follicle-specific epithelial keratins and *red* indicated epithelial keratins

The α-keratin gene family was further classified according to the phylogenetic relationship reconstructed using ML and Bayesian approaches. We found that almost all of the α-keratin genes of 16 species could be clustered into respective clades according to each subfamily, which can be regarded as orthologous genes in phylogenetic tree (Figs. 2 and 3). However, some genes within one specie grouped together rather than with the members of the same family in other species, such as among the genes of K31, K33A, K33B and K34 in type I, as well as the K6A, K6B, K6C, K81, K83, K87 and K86 in the type II (Figs. 2 and 3). This phenomenon suggested that such members within a repetitive family did not evolve independently of each other but under concerted evolution [13], which was also found in other mammals such as horse, dog, human, etc. [14]. We further used the RDP4 to test whether the gene conversion and recombination resulted in these 19 gene groups supposed to be under concerted evolution. The result showed that only three gene group supposed to be under concerted evolution was due to significant level of gene conversion and recombination (Additional file 2).

Species-specific evolution of α-keratin gene family was identified in the marine mammals, including differences in the total number of genes, functional genes, as well as pseudogenes. Nine keratin subfamilies were completely absent in cetaceans when compared to its terrestrial relative of artiodactylans, such as six type I genes (K10, K25, K27, K28, K33A, K33B) and three type II genes (K1, K73, K77) (Fig. 1). Most importantly, the cetacean species were also exclusively lost of 10 keratin genes (type I: K10, K25, K27, K28, K33A, K33B, K37; type II: K1, K73, K77) when compared with other marine mammals (Fig. 1). Besides these lost genes, we also examined 12 pseudogenes in cetaceans (type I: K9, K26, K34, K38, K39, K42; type II: K2, K6B, K74, K75, K79, K82, Fig. 1). Previous study has reported that deficient for K6B (epithelial keratins) may contributed to the absence of hair and nail in mice [28]. By contrast, mutations in K1 and K10 were shown to be associated with bullous congenital ichthyosiform erythroderma (BCIE) [29]. Collectively, cetaceans were exclusively lost K6B, K1 and K10, which is likely to contribute to its hair-loss phenotype to adapt to the completely aquatic environment. For complete aquatic manatee, however, we found that only four keratin subfamilies, such as K9, K6C, K6B, K39, were uniquely lost when compared with its terrestrial relatives, the elephant (Fig. 1).

When only cetaceans were considered, we found the average number of α-keratin genes in baleen whales was 43.5, slightly higher than that in toothed whales (averaged 34.8). Specially, pseudogenization rate of hair follicle-specific keratin genes was higher in toothed whales (42.86%) than in baleen whales (35.29%). The much higher number of intact functional keratin genes in the baleen whales may be associated with the presence of keratinized baleens.

### Sharing keratin genes loss and pseudogenes in cetaceans and manatee as response for convergent hair-less phenotype

Marine mammals are relatively independent in evolution but with similar phenotype changes and associated physiological features to adaptation to aquatic lifestyle. Remarkably, both cetaceans and sirenians are fully aquatic and therefore encompass convergent hair-less phenotype to adapt to aquatic environment, since the lack of fur improves their hydrodynamic and subaquatic movements. Cetaceans have apparent reduction of the α-keratin gene repertoire and increased rate of pseudogenization while the manatees have a comparable number of α-keratin and pseudogenization rate when compared to their respective terrestrial relatives. Importantly, both groups were found to share common gene loss in the four subfamilies, i.e. K39, K9, K42, K74 that play a key role in hair development, likely to be responsible for hair-less phenotype. For example, it has been reported that heterozygous mutations in K74 could cause autosomal dominant woolly hair, and/or hypotrichosis simplex [30] whereas mutations in K9 gene caused epidermolytic palmoplantar keratoderma, leading to manifests cytolysis and epidermal thickening [31]. In addition, K39 as a member of the hair-keratins, was identified to play a key role in late hair differentiation [32]. By contrast, when another components of hair, KRTAP, are considered, a significant gene number reduction (81 in manatee vs. 112 in elephant) and increased pseudogenization rate (41.98% in manatees vs. 20% in elephant) were detected in manatee. Similarly, it has been recently reported that dolphins only have 35 KRTAP genes (compared to 145 genes in the cow) but with highest pseudogenization rate (74% relative to the 19% of mammalian average) [13]. However, we have detected average of 65 functional KRTAP genes in the pinnipeds that have abundant hair, which were comparable to that of terrestrial relatives, such as panda with 64 KRTAP genes [13]. Collectively, a reduced number of functional KRTAP genes, high percentage of KRTAP pseudogenes, and common keratin gene loss in both cetaceans and manatees might be responsible for their convergent hair-less phenotype.

## Conclusion

In this study, we provide a comprehensive characterization of α-keratin genes among marine mammals and shed light on the mechanisms involved in the evolution of this gene family. Our results show an apparent reduction in the α-keratin gene repertoire and increased rate of pseudogenization in cetaceans when compared to terrestrial counterparts, which may be associated with their hairless phenotype. In contrast, the evolution pattern of α-keratin genes in pinnipeds was comparable to that of their respective terrestrial relatives, which is well matched with its fur coat. Interestingly, we found a reduced number of functional KRTAP genes, high percentage of KRTAP pseudogenes, and common keratin gene loss in both cetaceans and manatees, which might be as a result of convergent hair-loss phenotype to inhabit completely aquatic environment. Species-specific evolution of α-keratin gene repertoire identified in marine mammals was likely to contribute to their hair diversities phenotype although their life or part inhabiting in aquatic environment. Our study first indicated the genetic basis of hair diversities of marine mammals.

## Additional files

Additional file 1: The excel file shows the genomic coordinates of the α-keratin gene repertoires in 16 mammalian species. (XLS 98 kb)

Additional file 2: Results of RDP4 showing unique recombination and conversion events with statistical significance *p* value of less than 0.01. (XLS 11 kb)

## Abbreviations

BCIE: Bullous congenital ichthyosiform erythroderma
EIF1: Eukaryotic translation initiation factor 1
EIF4B: Eukaryotic translation initiation factor 4B
FAIM2: Fas apoptotic inhibitory molecule 2
HGT: High glycine-tyrosine
HS: High/ultrahigh cysteine
KRTAPs: Keratin associated-proteins
ML: Maximum likelihood
SMARCE1: SWI/SNF related, matrix associated, actin dependent regulator of chromatin, subfamily e, member 1
